# How the New Type of Entrepreneurship Education Complements the Traditional One in Developing Entrepreneurial Competencies and Intention

**DOI:** 10.3389/fpsyg.2019.02048

**Published:** 2019-09-13

**Authors:** Shu-Mei Wang, Hsiu-Ping Yueh, Pei-Chang Wen

**Affiliations:** ^1^Department of Bio-Industry Communication and Development, National Taiwan University, Taipei, Taiwan; ^2^Department of Psychology, National Taiwan University, Taipei, Taiwan; ^3^Chung-Hua Institution for Economic Research, Taipei, Taiwan

**Keywords:** entrepreneurship education, business and management education, entrepreneurial competency, entrepreneurial intention, higher education

## Abstract

While business schools aim to train students to develop specialized professional competencies, knowledge, and skills related to management and corporate functions according to their major programs, entrepreneurship education in higher education intends to develop students’ entrepreneurial competencies and intention. However, the entrepreneurial and managerial domains are not mutually exclusive but overlap to a certain extent. This study utilized the National Taiwan University (NTU) as a case to explore the effects of two paths of entrepreneurial education at NTU on the development of students’ entrepreneurial competencies and intention. The aim of this study was to investigate differences in business school students’ entrepreneurial competencies and intention between those who took the Creativity and Entrepreneurship Program (CEP) and those who did not, and to explore the context limits or facilitations in the entrepreneurship education of college students in different academic disciplines of management school. Results of the study showed that the CEP course did have positive impacts on all entrepreneurial competencies and intention, that the effectiveness on the attitude domains was more evident than that on the knowledge or skills domains, and that academic disciplines did have a context effect on students’ entrepreneurial competencies and intention. This study sheds further light on the “black box” of context limits or facilitations in entrepreneurship education. Implications of the study are that it may lead to a complementary framework of effectively integrating the entrepreneurial program with the business and management courses, which would better facilitate students’ learning of entrepreneurship competencies and may increase their intention to become future entrepreneurs.

## Introduction

Entrepreneurship has been one of the most potent economic forces in the world for the past few decades (Kuratko, 2005; Aparicio et al., 2016). Due to the high levels of unemployment amongst young people, youth entrepreneurship has also gained attention as a way to foster employment opportunities. Therefore, many studies in the literature address the importance of entrepreneurial education in colleges and try to explore factors that affect entrepreneurial intention across disciplines, including business and management schools (i.e., Kaijun and Sholihah, 2015; Gelaidan and Abdullateef, 2017; Hockerts, 2017). However, the development of a method of actually assessing the effects and the impact of entrepreneurship programs has increased in importance, and methods to clarify their relationships to management education deserve more research attention.

While a knowledge base is one major aspect of context, some researchers have investigated how context limits or facilitates the effects of entrepreneurial education. Previous studies have shown that the effects of entrepreneurial education on strengthening entrepreneurial intention are dissimilar in various disciplines (i.e., Iwu et al., 2016; Maresch et al., 2016; Refai and Klapper, 2016). Meanwhile, entrepreneurship education is deemed as a different pedagogy from those in typical business education and management training. Debate continues on the future of the business and management schools (Pfeffer and Fong, 2002; Kaplan, 2018) for the rising tides of youth entrepreneurship. One radical realignment suggests a move away from conventional wisdom and toward a constructivist framework that emphasizes the social aspects of learning in entrepreneurship education (Rae, 1997; Kirby, 2004; Löbler, 2006). From the perspective of education system design, such a move is neither necessary nor practical, and both enterprise management and start-up development are equally important to the economy.

As Kuratko (2005) contended, a core objective of entrepreneurship education is that it is different from business and management education. Business and management education emphasize the operation of an ongoing business (Gartner and Vesper, 1994), whereas entrepreneurial education must address business entry (Gartner et al., 1992). While students of business schools are trained in specialized professional competencies, knowledge, and skills related to management and corporate functions according to their major programs, they may not necessarily learn entrepreneurship during their academic training. In fact, as Ireland et al. (2003) argued, because the entrepreneurial and managerial domains are not mutually exclusive but overlap to a certain extent, it might be more effective to integrate management school courses and entrepreneurship programs into a more complementary education framework to cultivate talents in enterprise management and start-up development with a minimum restructuring of the education system.

Previous studies have tried to compare the effects of students’ professions on their knowledge and intention to become entrepreneurs. However, few researchers have explored the effects of students in different programs of business and management schools on their performance and attitude toward entrepreneurship. The aim of this current study was to explore the context effects, including various academic disciplines of typical business school training and an entrepreneurial program, on students’ learning of entrepreneurship and their entrepreneurial intention.

The research aimed to answer the following research questions: (1) Are there differences in students’ entrepreneurial competencies between those who minored in an entrepreneurship program and those who did not? (2) Are there differences in students’ entrepreneurial intention between those who minored in an entrepreneurship program and those who did not? (3) Are there differences in students’ entrepreneurial competencies among students in different majors in management school, including Accounting, Business Administration, Finance, and International Business? (4) Are there differences in students’ entrepreneurial intention among students in different majors of management school, including accounting, business administration, finance, and international business?

It is anticipated that this study will shade a light on the “black box” of context limits or facilitations in the entrepreneurship education of college students. It is expected to lead to a complementary framework for effectively integrating the entrepreneurial program with business and management courses, which would better facilitate students’ learning of entrepreneurship competencies and may increase their intention to become future entrepreneurs.

## Literature Review

### Competency Development in Business and Management Education

The world’s first business school, ESCP Europe, was established in 1819 in Paris, France. Since its founding, the world has seen the development of various types of business schools, such as schools of business, business administration, and management. Kaplan (2018) proposed a model called the “four corners of a building analogy” to categorize the roughly 13,000 business schools worldwide with continuums into eight types. The four criteria/corners are culture (Europe–United States), compass (international/global–regional/local), capital (public–private), and content (teaching–research). While many business schools typically teach courses in areas such as entrepreneurship, finance, marketing, and strategy, many also offer highly specialized programs such as executive education programs (MBA, EMBA, Global MBA) and innovation and entrepreneurship programs. In addition, they often adopt accreditation systems to monitor their quality assurance of business and management.

According to the American Assembly of Collegiate Schools of Business (AACSB), one of the main accreditation agencies for business schools in the United States, the educational outcomes provided by business schools can be grouped into two categories: (1) content and (2) skills and personal characteristics (AACSB International Accreditation Coordinating Committee and AACSB International Accreditation Quality Committee, 2013). The content category comprises seven knowledge areas of the core courses offered by business schools, as follows: accounting, business environment and strategy, finance, human resources and organization theory, marketing, management information systems, quantitative analysis/operations research/production, and operation management. As for “learning and teaching,” the AACSB, 2018 business accreditation standards (update of 2013 version) indicate that all general management and specialist degree programs at the bachelor’s, master’s, and doctoral levels should normally include learning experiences that address the following general skill areas and general business and management skill areas. General skill areas include written and oral communication, ethical understanding and reasoning, analytical thinking, interpersonal relations and teamwork, diverse and multicultural work environments, reflective thinking, application of knowledge, and integration of real-world business experiences. General business knowledge areas include economic, political, regulatory, legal, technological, and social contexts of organizations in a global society; social responsibility, including sustainability, diversity and ethical behavior; approaches to management, financial theories, analysis, reporting, and markets; systems and processes in organizations, including planning and design, production/operations, supply chains, marketing, and distribution; group and individual behaviors in organizations and society; and other specified areas of study related to concentrations, majors, or areas of emphasis (AACSB Business Accreditation, 2019).

Although many accreditation institutions have defined a rather broad range of knowledge and skills for business schools to teach, they have tended to offer a rather general approach, as Kaplan (2018) argued. Many researchers have also proposed competencies that business and management education should ensure that students possess, such as leadership, oral communication/presentation skills, written communication, planning and organizing, information gathering and problem analysis, decision making, delegation and control, self-objectivity, and disposition to lead. Moreover, the continued fragmentation of business education into ever narrower specializations (Zeithaml and Rice, 1987) enlarges the gaps in the core competencies between the departments of business and management colleges. Therefore, whether business schools can really ensure that students learn the necessary business and management knowledge and skills and possess the requisite competencies, and especially whether they can apply what they have learned in real business contexts, remains controversial and demands more research investigations.

### Entrepreneurial Competency and Intention Development in Entrepreneurship Education

As noted above, most business schools around the world are running specific programs such as innovation and entrepreneurship programs. Entrepreneurship education in higher education intends to develop students’ entrepreneurial competencies and intention (Souitaris et al., 2007). Entrepreneurial competencies are argued to have impacts on the willingness and capability to perform the entrepreneurial tasks of new value creation, and they could be used as predictors of business success (Kraiger et al., 1993; Fisher et al., 2008; Sánchez, 2011; Lackéus, 2015).

To define entrepreneurial competencies, the three domains that must be considered are knowledge, skills, and attitudes. The knowledge domain, considered the basis of entrepreneurial competency, includes basics of entrepreneurship, value creation, idea generation, opportunities, accounting, finance, technology, marketing, risk, and other key concepts (Kraiger et al., 1993). As for entrepreneurial skills, Fisher et al. (2008) defined a framework for analyzing learning outcomes in entrepreneurship education based on the marketing, resource, opportunity, interpersonal, learning, and strategic skills that are useful in the entrepreneurial process. Literature contributing to the linkage between entrepreneurial education and the entrepreneurial attitudes identifies the following themes of attitudes as positive: entrepreneurial passion/inspiring entrepreneurial passion (Fisher et al., 2008), entrepreneurial identity/believing in entrepreneurial value (Krueger, 2005, 2007), innovativeness/novel thoughts and actions (Krueger, 2005; Murnieks, 2007), and self-insight/knowledge of personal fit with being an entrepreneur (Kraiger et al., 1993).

Lackéus (2015) further proposed a classification of entrepreneurial competencies based on the framework constructed by Fisher et al. (2008). This classification categorized eleven themes of entrepreneurial competencies grouped into three domains (see Table 1). In this study, this framework was used to develop our measures of students’ entrepreneurial competencies.

**TABLE 1:** The classification of key entrepreneurial competencies.

**Domain**	**Themes**	**Interpretation**
Knowledge	Basic business knowledge	Basics of entrepreneurship, value creation, idea generation, opportunities, accounting, finance, technology, marketing, risk, etc.
Skills	Marketing skills	Conducting market research, assessing the marketplace, marketing products and services, persuasion, getting people excited about your ideas, dealing with customers, communicating a vision
	Resource skills	Creating a business plan, creating a financial plan, obtaining financing, securing access to resources.
	Opportunity skills	Recognizing and acting on business opportunities and other kinds of opportunities, product/service/concept development skills
	Interpersonal skills	Leadership, motivating others, managing people, listening, resolving conflict, socializing
	Learning skills	Active learning, adapting to new situations, coping with uncertainty
	Strategic skills	Setting priorities (goal setting) and focusing on goals, defining a vision, developing a strategy, identifying strategic partners
Attitudes	Entrepreneurial passion	“I want.” Need for achievement
	Entrepreneurial identity	“I am/I value.” Deep beliefs, role identity, values
	Innovativeness	“I create.” Novel thoughts/actions, unpredictable, radical change, innovative, visionary, creative, rule breaker
	Self-insight	Knowledge of personal fit with being an entrepreneur/being entrepreneurial

*Adapted and modified from Lackéus (2015).*

On the other hand, entrepreneurial intention is considered as a personal orientation to start a business, become self-employed, or lead venture creations. Entrepreneurial intention is the key element in understanding entrepreneurship for the reason that creating a new business is a planned behavior (Bird, 1988). Many studies have investigated entrepreneurial intention utilizing the Theory of Planned Behavior (TPB) (i.e., Krueger et al., 2000; Souitaris et al., 2007; Karimi et al., 2013; Kaijun and Sholihah, 2015) and found that intention is the best predictor of planned behavior. Entrepreneurial intention is considered as an antecedent of entrepreneurship, and the determinants of entrepreneurial intention are well explored (Saeed et al., 2015; Yukongdi and Lopa, 2017).

Based on the TPB framework, previous studies have explored the impact of education on entrepreneurial intention. Entrepreneurship education is considered to be one of the key instruments for increasing entrepreneurial intention, and many researchers have confirmed the positive linkage between entrepreneurship education and entrepreneurial intentions (i.e., Souitaris et al., 2007; Zhang et al., 2014; Kaijun and Sholihah, 2015; Rauch and Hulsink, 2015; Westhead and Solesvik, 2016).

However, some previous studies do not support the positive relationship between entrepreneurial education and entrepreneurship intention (Oosterbeek et al., 2010). The contextual differences, such as those resulting from a knowledge base either from entrepreneurship education or personal professions, should be taken into consideration as deceptive sources (Martin et al., 2013; Maresch et al., 2016). To education system designers, both potential sources of contextual differences are equally important and worthy of further investigation. The current study intended to test the effects of an entrepreneurial education program and of the academic disciplines of business schools on students’ entrepreneurial competencies and intention to become entrepreneurs.

## Methods

### The Context: Management Education and Creativity and Entrepreneurship Program at NTU

This study utilized the National Taiwan University (NTU) as a case to explore the effects of two paths of entrepreneurial education at NTU on students’ entrepreneurial competencies and intention development. The management education of NTU was first founded in 1948. As of today, the college of management of NTU provides leading business programs in Taiwan and was ranked 48th by the ESSEC Business School in 2018 (ESSEC Business School, 2018). It consists of five departments for undergraduates: Accounting, Business Administration, Finance, International Business, and Information Management. Except for the department of Information Management,^1^ these departments are considered to be representative of typical management education disciplines.

As shown in Table 2, the capabilities of the core courses of the four departments of the college of management at NTU consist mostly of knowledge and skills orientation, in accordance with their academic disciplines. All courses in the department of finance can be grouped into solely professional skills, while there are five groups of professional skills among the courses of the department of international business. Other than knowledge and skills, the abilities to lead or execute teamwork are provided by courses of three of the departments, all but the department of accounting. The ability to identify and solve problems and the ability to innovate are solely provided by the department of business administration. With noticeable variations among the course attributes of the four departments of the college of management, it is reasonable to expect students from these four departments to be equipped with different knowledge bases according to their majors. Meanwhile, students’ abilities of leadership, problem-solving and innovation are anticipated to be unevenly distributed among the four departments due to disparities in the courses.

**TABLE 2:** Capabilities of core courses of four departments of the college of management, NTU.

	**Capabilities: number of core course (% of all courses)**			
	
**Capabilities**	**Accounting number of courses: 89**	**Business Administration number of courses: 101**	**Finance number of courses: 97**	**International Business number of courses: 98**
Knowledge	• Basic business knowledge: 24 (27%) • Humilities/legal knowledge: 20 (22%)	• Knowledge of industry environment and development: 44 (44%) • Abidance of professional ethics: 27 (27%)	• Financial ethics and social responsibility: 45 (46%)	•Theoretical foundations of business management: 30 (31%)
Language and communication skills	• Language and communication skills: 33 (37%)	• Oral and written presentation and communication skills: 68 (67%) • Global perspectives and foreign language ability: 35 (35%)	• Global view and proficiency in foreign language: 42 (43%)	(None)
Professional skills	• Professional accounting knowledge: 46 (52%) • Management knowledge and ability: 29 (33%) • Information analysis and application ability: 11 (12%)	• Professional knowledge and skills: 90 (89%) • Ability in the applications of analytical and quantitative tools: 54 (53%)	• Masterful in financial theory and practice: 91 (94%)	• Application of financial management and economics: 44 (45%) • International business management capability: 41 (42%) • International branding and marketing capability: 27 (28%) • Industrial analyzing capability: 26 (27%) • Quantitative logical thinking and analyzing capability: 20 (20%)
Ability to lead or for teamwork	(None)	• Ability for teamwork: 58 (57%) • Ability to lead: 24 (24%)	• Ability to work as a team member: 51 (53%)	• The awareness of teamwork and social responsibility: 24 (24%)
Ability to identify and solve problems	(None)	• Ability to identify and solve problems: 91 (90%)	(None)	(None)
Ability to innovate	(None)	• Ability to innovate: 46 (46%)	(None)	(None)

*Authors, compiled from course maps of each department of the management college of NTU.*

On the other hand, the Creativity and Entrepreneurship Program (CEP) at NTU was initiated in 2008, so it is 60 years younger than those of the traditional management education. CEP is designed to act as an entrepreneurial platform program to recruit students who aspire to become entrepreneurs or who would like to understand entrepreneurship, and it provides them with instruction and genuine hands-on experience. Through cross-disciplinary orientation courses in active learning and participation classrooms, the learning path of CEP intends to motivate students to try entrepreneurial thinking and engage in cooperative activities. One featured learning activity of core CEP courses is the opportunity for interaction between students and experienced entrepreneurs from the real business world.

As for the courses offered by CEP, it is very different from the courses of the departments of the college of management. It is designed to provide opportunities for students to actively participate in learning and to accumulate experience from practical exercises. Despite course rotations among semesters, the spirit of CEP courses is to equip enrolled students with teamwork capabilities and problem-solving skills. Most CEP courses can be characterized as problem-oriented, theme-based, and hands-on learning activities. The learning pattern of CEP requires students to mobilize all their capabilities to solve specific issues or problems through teamwork practice. It therefore provides connectivity and convergence of the knowledge and professional skills they have acquired in their regular departmental courses. Students enrolled in CEP are requested to draw up business plans with real market pain points, on their own or in a group. Throughout the journey of CEP courses, students are expected to be motivated to transfer their learnt skills, fragmented by the course subjects of their departments, into experience by exercising problem solving on their proposed business plans.

### Research Participants

Purposeful sampling was used to recruit participants from students enrolled in the four departments of the college of management, including both students who had taken and those who had not taken CEP courses. Respondents were fully informed about the research purpose on the questionnaire, and they returned the questionnaires voluntarily. Per applicable institutional and national guidelines, no additional consent approval was required, as all respondents were voluntary and anonymous. In total, 267 fully completed questionnaires were returned. As seen in Table 3, the descriptive analysis of the sample showed well-balanced sampling among genders, CEP taken or not, and departments of the college of management.

**TABLE 3:** Descriptive analysis of sample.

**Variable**		***n* = 267**	
	
	**Frequency**		**%**
**Gender**			
Female	139		52.1
Male	128		47.9
**Class**			
Freshman	21		7.9
Sophomore	37		13.9
Junior	98		36.7
Senior	111		41.5
**CEP-taken**			
Yes	126		52.8
No	141		47.2
**Department**			
Accounting	65		24.3
CEP-taken-Yes		31	
CEP-taken-No		34	
Business Administration	65		24.3
CEP-taken-Yes		31	
CEP-taken-No		34	
Finance	68		25.5
CEP-taken-Yes		30	
CEP-taken-No		38	
International Business	69		25.8
CEP-taken-Yes		34	
CEP-taken-No		35	

### Measures

In this study, the classification of entrepreneurial competencies proposed by Lackéus (2015) with entrepreneurial intention was adopted to compose the survey measures (see Appendix). The questionnaire consisted of 20 items, to which participants responded on a six-point Likert-type scale (ranging from 1 = strongly disagree to 6 = strongly agree). The collected data were analyzed in SPSS for Windows version 22.

## Results and Discussion

**FIGURE 1:**
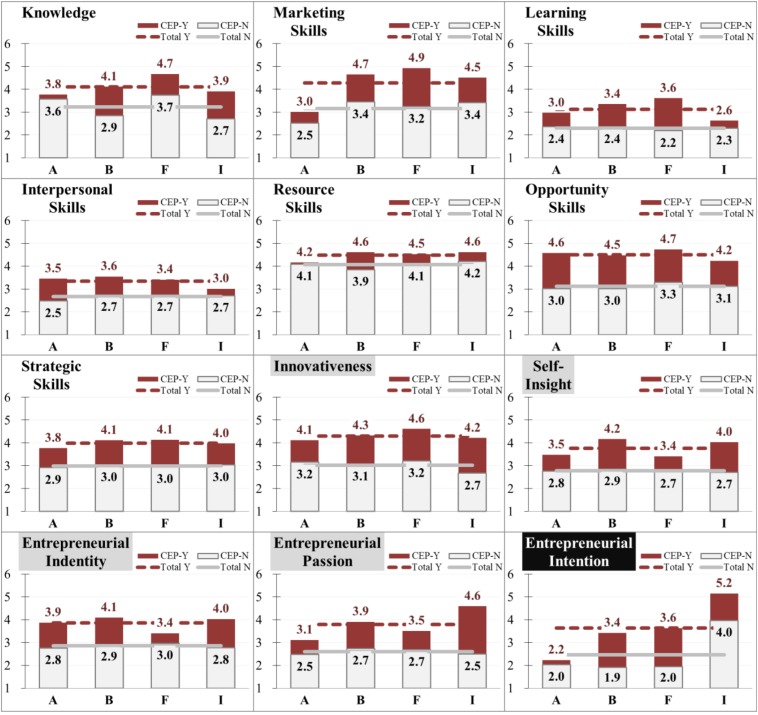
Comparisons of entrepreneurial competencies and intention among samples. Department A: Accounting; Department B: Business Administration; Department F: Finance; Department I: International Business. Scale measurements of which ranging from 1 (strongly disagree) to 6 (strongly agree) out of a six-point Likert-type scale.

To answer the research question and objectives, an independent-sample *t*-test was conducted to compare CEP-taken (CEP-Y) and CEP-not-taken (CEP-N) for each student group of the departments of the college of management. The integrated results are shown in Table 4, and the significance of score differences is noted in the “Diff” column. The means of each entrepreneurial competency and intention by sample groups are shown in Figure 1 to visualize the comparisons. Table 5 summarizes the test results of entrepreneurial competencies and intention by department among the sample.

**TABLE 4:** The differences in entrepreneurial competencies and intention by CEP-taken or not-taken among samples.

**Department**	**Total**					**Accounting**					**Business Administration**					**Finance**					**International Business**				
	**CEP-N**		**CEP-Y**		**Diff.**	**CEP-N**		**CEP-Y**		**Diff.**	**CEP-N**		**CEP-Y**		**Diff.**	**CEP-N**		**CEP-Y**		**Diff.**	**CEP-N**		**CEP-Y**		**Diff.**
**Competencies**	**Mean**	**SD**	**Mean**	**SD**		**Mean**	**SD**	**Mean**	**SD**		**Mean**	**SD**	**Mean**	**SD**		**Mean**	**SD**	**Mean**	**SD**		**Mean**	**SD**	**Mean**	**SD**	
Knowledge	3.23	1.00	4.10	1.37	^∗∗^	3.56	0.99	3.77	1.36		2.85	0.96	4.11	1.35	^∗∗^	3.74	0.76	4.67	1.56	^∗∗^	2.71	0.93	3.91	1.11	^∗∗^
Marketing skills	3.15	0.96	4.27	1.47	^∗∗^	2.51	0.75	3.01	1.24		3.44	0.99	4.65	1.20	^∗∗^	3.24	0.88	4.93	1.60	^∗∗^	3.41	0.95	4.51	1.08	^∗∗^
Resource skills	4.06	1.14	4.48	1.20	^∗^	4.09	1.24	4.16	1.16		3.85	1.28	4.61	1.38	^∗∗^	4.08	1.11	4.53	0.90		4.21	0.93	4.62	1.28	
Opportunity skills	3.11	0.89	4.50	1.12	^∗∗^	3.01	0.85	4.58	1.26	^∗∗^	3.01	0.98	4.48	1.06	^∗∗^	3.29	0.95	4.73	1.02	^∗∗^	3.11	0.76	4.24	1.13	^∗∗^
Interpersonal skills	2.67	0.96	3.34	1.20	^∗∗^	2.47	0.96	3.45	1.18	^∗∗^	2.74	0.90	3.55	1.18	^∗∗^	2.74	1.06	3.41	1.38	^∗^	2.71	0.92	3.01	1.04	
Learning skills	2.29	0.85	3.12	1.38	^∗∗^	2.35	0.92	2.97	1.48	^∗^	2.35	0.81	3.35	1.45	^∗∗^	2.18	0.90	3.61	1.32	^∗∗^	2.29	0.79	2.62	1.10	
Strategic skills	2.98	1.09	3.99	1.28	^∗∗^	2.91	1.06	3.77	1.36	^∗∗^	3.03	1.14	4.11	1.33	^∗∗^	2.95	1.13	4.13	1.33	^∗∗^	3.03	1.07	3.97	1.14	^∗∗^
Entrepreneurial passion	2.60	1.11	3.79	1.53	^∗∗^	2.47	0.99	3.11	1.56		2.74	1.24	3.91	1.45	^∗∗^	2.68	1.06	3.51	1.71	^∗∗^	2.49	1.15	4.59	1.02	^∗∗^
Entrepreneurial identity	2.86	0.87	3.86	1.13	^∗∗^	2.76	0.92	3.87	1.61	^∗∗^	2.91	0.91	4.09	1.38	^∗∗^	2.97	0.85	3.41	1.30		2.77	0.81	4.03	1.17	^∗∗^
Innovativeness	3.02	0.94	4.30	1.01	^∗∗^	3.15	0.96	4.11	0.98	^∗∗^	3.06	0.89	4.32	1.19	^∗∗^	3.21	1.04	4.61	0.89	^∗∗^	2.66	0.77	4.21	0.93	^∗∗^
Self-insight	2.77	0.95	3.75	1.22	^∗∗^	2.76	0.92	3.48	1.15	^∗∗^	2.85	0.93	4.16	1.10	^∗∗^	2.74	1.06	3.41	1.38	^∗^	2.71	0.93	4.03	1.17	^∗∗^
Entrepreneurial intention	2.46	1.48	3.64	1.67	^∗∗^	2.03	1.11	2.23	1.12		1.91	1.14	3.42	1.34	^∗∗^	1.95	1.14	3.63	1.85	^∗∗^	3.97	1.40	5.15	0.74	^∗∗^

*Scale measurements of which ranging from 1 (strongly disagree) to 6 (strongly agree) out of a six-point Likert-type scale. Posteriori test: LSD. ^∗^*P* < 0.05. ^∗∗^*P* < 0.01.*

**TABLE 5:** Test results of entrepreneurial competencies and intention by departments among samples.

**Competencies/Departments**	**CEP chosen-yes**						**CEP chosen-not**					
	**A vs. B**	**A vs. F**	**A vs. I**	**B vs. F**	**B vs. I**	**F vs. I**	**A vs. B**	**A vs. F**	**A vs. I**	**B vs. F**	**B vs. I**	**F vs. I**
Knowledge		^∗∗^F > A				^∗∗^F > I	^∗^B > A		^∗∗^A > I	^∗∗^F > B		^∗∗^F > I
Marketing skills	^∗∗^B > A	^∗∗^F > A	^∗∗^I > A				^∗∗^B > A	^∗∗^F > A	^∗∗^I > A			
Resource skills												
Opportunity skills						^∗^F > I						
Interpersonal skills						^∗^F > I						
Learning skills		^∗^F > A		^∗∗^F > B	^∗∗^B > I	^∗∗^F > I						
Strategic skills												
Entrepreneurial passion		^∗^F > A	^∗∗^I > A		^∗^I > B	^∗∗^I > F						
Entrepreneurial identity				^∗∗^B > F		^∗^I > F						
Innovativeness		^∗^F > A							^∗^A > I			^∗^F > I
Self-insight	^∗^B > A		^∗^I > A	^∗^B > F		^∗^I > F						
Entrepreneurial intention	^∗∗^B > A	^∗∗^F > A	^∗∗^I > A		^∗∗^I > B	^∗∗^I > F			^∗∗^I > A		^∗∗^I > B	^∗∗^I > F

*Posteriori test: LSD. Department A: Accounting; Department B: Business Administration; Department F: Finance; Department I: International Business. ^∗^*P* < 0.05. ^∗∗^*P* < 0.01.*

### The CEP-Y Students Showed Significantly Higher Scores on Entrepreneurial Competencies and Intention Than the CEP-N Students Did

As seen in Table 4, almost every comparison presented a high level of significance between the CEP-Y and CEP-N groups. These results suggested that CEP courses really did have significant effects on entrepreneurial competencies and intention with regard to every competency of all the respondents of one group “Total,” as well most competencies of the CEP-Y and CEP-N groups of each department of the College of Management, NTU. In general, the CEP-Y students scored significantly higher on entrepreneurial competencies and intention than did their CEP-N counterparts.

Among all, the sample from the department of business administration showed full consistency in the significance of CEP-taken-or-not in all competencies and intention, while students of the department of accounting showed the fewest significant results out of the CEP-taken-or-not tests on competencies. These results suggest that the bodies of knowledge and skills of the four departments might diversify the effectiveness of CEP courses on entrepreneurial competencies and intention, as will be discussed in section “Diverse Effectiveness of CEP Courses on Entrepreneurial Competencies Among Four Departments of the College of Management.”

### CEP Affects Attitudes and Intention More Than It Does Knowledge and Skills

As Figure 1 shows, between the CEP-Y and CEP-N groups, greater differences were found in the attitude domains and entrepreneurial intention than in competencies of knowledge and skills. This finding might be related to the self-selectiveness of CEP-Y students, who aspire to be entrepreneurs or would like to learn about entrepreneurship. However, it is irrefutable that, through CEP courses featuring problem/issue-oriented and hands-on activities, the new type of entrepreneurship education contributes to inspiring students and possibly leads to higher self-assessments on the scales of the attitude domains and entrepreneurial intention.

### Diverse Effectiveness of CEP Courses on Entrepreneurial Competencies Among Four Departments of the College of Management

#### CEP Courses Are Less Effective on Enhancing the Entrepreneurial Competencies and Intention of Students of the Department of Accounting

It is notable that five domains in Table 4 had insignificant gaps of entrepreneurial competencies and intention according to CEP-Y or CEP-N in the sample of the department of accounting. Their self-reported scales of entrepreneurial intention and passion were mostly the lowest of the four departments, whether they were in the CEP-N or CEP-Y group of the department of accounting. In addition, the differences in entrepreneurial intention and passion of the CEP-Y and CEP-N groups were the smallest among all the departments, as can easily be seen in Figure 1. For the knowledge, marketing skills and learning skills, similar results were found. For students of the department of accounting, it seemed that CEP courses were less effective on enhancing entrepreneurial competencies and intention, as compared with students in the other departments of the college of management.

#### CEP Courses Highly Improved the Self-Assessed Scores of Several Skills of the Students of the Department of Finance

Figure 1 illustrates that the scores of the students of the department of finance on many entrepreneurial skills and intention were higher in the CEP-Y group than in the CEP-N group, while minimum scale gaps on attitudes such as self-insight and entrepreneurial identity were noted. This was a dissimilar pattern from the general finding that CEP impacted on attitudes more than on skills. Students of the department of finance indicated that CEP courses highly improved their self-assessed scores of some skills, such as marketing, learning and strategic skills. The difference in entrepreneurial intention of the CEP-Y and CEP-N groups was also the largest among the four departments. It is reasonable to argue that CEP courses worked better on score improvements of these skills for students of the department of finance than they did for students of other departments of the college of management.

#### CEP Courses Provided Well-Balanced Enhancement of Self-Assessed Scores of All Entrepreneurial Competencies and Intention for the Students of the Department of Business Administration

Comparing the CEP-Y and CEP-N groups of the department of business administration, significant differences existed in each test result of the score gaps of entrepreneurial competencies and intention. It can be concluded that the CEP courses provided well-balanced enhancement of the self-assessed scales of all entrepreneurial competencies in the domains of knowledge, skills and attitudes, and intention to students of the department of business administration. The most comprehensive increases in entrepreneurial competencies suggested that the students of the department of business administration were the greatest beneficiaries of the CEP courses among the four departments of the college of management.

#### CEP Courses Increased the Self-Assessed Scores of Entrepreneurial Attitudes and Intention More Than They Did the Knowledge and Skills of the Students of the Department of International Business

For the groups in the department of international business, all differences in entrepreneurial attitudes and intention were highly significant for the CEP-Y and CEP-N groups, but a few differences in the skills domains were found to be insignificant, namely, the resource skills, interpersonal skills and learning skills. The highest scores of the entrepreneurial intention of CEP-N students were considerably lower than those of the CEP-Y students of the department of international business. This result could be taken as positive support for the CEP courses as they significantly reinforce the entrepreneurial intention of the enrolled students, regardless of how high the scores of their CEP-N counterparts were.

### Comparisons of Four Departments of the College of Management

#### CEP Courses Amplified Differences in Entrepreneurial Intention Among Departments

Table 5 presents comparisons among the samples for all CEP-Y and CEP-N students. This table helps to clarify the context issues with better understanding of the relative relationships of the self-assessed scores of entrepreneurial knowledge, skills, and attitudes between any two departments.

For both CEP-N and CEP-Y students, those in the department of international business always showed stronger entrepreneurial intention than those in other departments. In the other three departments, students’ entrepreneurial intentions revealed more cases of significant differences only for CEP-Y students. Moreover, students in the department of accounting had the lowest self-assessed scores of entrepreneurial intention of all CEP-Y students. It seemed that the CEP courses amplified the differences in entrepreneurial intention among the four departments of the college of management. The CEP-Y students of the department of international business had the highest self-assessed scores of entrepreneurial intention among the departments, while those of the department of accounting had the lowest.

#### CEP Courses Created Scale Gaps Among the Departments in Attitudes More Than in Knowledge and Skills

For all CEP-N students, the self-assessments of the majority of the skills domains had no significant differences among the four departments. Only on marketing skills were the self-assessed scores of students of the department of accounting significantly lower than those of any of the other departments. Another domain with significant differences among departments was that of knowledge; the self-assessed scores were in the order of the department of finance, then the department of business administration, then the department of accounting, and finally the department of international business.

As for CEP-Y students, although the department of accounting had the lowest self-assessed scores of marketing skills, the order of scales of knowledge was not as clear as that of CEP-N students. Similarly, to those of the CEP-N students, the self-assessed scores of knowledge of the CEP-Y students of the department of finance were significantly higher than those of the departments of accounting and international business. However, learning skills was revealed to be another domain with significant gaps among departments; the scores of the department of finance were higher than those of all other departments.

Therefore, CEP courses significantly increased scale gaps in the knowledge and skills domains among the departments. For the attitude domains, comparing results presented another pattern among departments. For all CEP-N students, innovativeness was the only attitude domain in which significant differences could be found among the departments. Yet for all CEP-Y students, significant differences among departments existed in all attitude domains. Although the order of the departments could not easily be determined, it was noted that the department of international business tended to have higher scores on attitude than did the other departments, while the departments of accounting and finance more frequently had lower scores as compared with the other two departments. As a result, it can be concluded from the above observations that the CEP courses affected the scale gaps of the departments more in the attitude domains than in the knowledge and skills domains.

## Conclusion

The aim of this study was to contribute knowledge on the context issue based on the effects of an entrepreneurial program. To be specific, it was expected that differences in business school students’ entrepreneurial competencies and intention would be found between those who had taken an entrepreneurship program and those who had not. This study also explored the context limits or facilitations in the entrepreneurship education of college students in different academic disciplines of a management school. The findings of this study have theoretical and practical implications as follows.

### CEP Courses Showed Effectiveness on All Entrepreneurial Competencies and Intention

The results of this study showed significantly higher gains in entrepreneurial competencies and intention in the CEP-Y groups than in the CEP-N groups of the same departments of the college of management, regardless of which one. The score differences in the attitude domains and entrepreneurial intention between groups were greater than those of the knowledge and skills domains. Both the self-selectiveness of CEP-Y students and the CEP courses, featuring problem/issue-oriented and hands-on activities, contributed to the results. This finding reconfirmed that CEP courses were successful in effectively improving students’ self-assessments of their entrepreneurial competencies and intention, as the program was designed to do.

### The Effectiveness of CEP Courses Was More Evident in the Attitude Domains Than in the Knowledge or Skills Domains

In general, the results of this study demonstrated the higher effectiveness of the CEP courses in the attitude domains than in the knowledge or skills domains. This finding was not unexpected, for CEP was designed to facilitate the accumulation of experiences and to provide both connectivity and the convergence of knowledge and professional skills acquired from the courses of the departments. In the unique learning pattern of the CEP courses, CEP-Y students were continuously motivated and encouraged by invited lecturers; entrepreneurs and participants in the start-ups eco-systems shared their knowledge of real market dynamics and exchanged ideas with the CEP students. Although some classes of a typical business school would also provide this kind of connectivity, they would not provide it as significantly as the CEP program course did, which was designed with this central feature.

### Bodies of Learned Knowledge or Future Outlooks on Employment and Rewards Might Contribute to the Dissimilar Patterns of Students of the Department of Finance

However, it can be concluded that the positive impacts of the new type of entrepreneurial program on promoting entrepreneurial competencies or entrepreneurial intentions do not equally benefit the students of the four departments of the college of management. This point raises the context issue, for students are equipped with different bodies of knowledge on corporate management, depending on their majors.

For example, a dissimilar pattern was found for students of the department of finance, for whom the effectiveness of the program was stronger in the knowledge and skills domains than in the attitude domains. This case has two possible explanations. First, the single dimension of professional skills of the course map of the department of finance suggested that students were equipped with a greatly focused body of professional knowledge and skills, which were further consolidated and better appraised through CEP courses. Second, future outlooks or expectations for employment and rewards of the graduates of the department of finance were less vulnerable, which could have led to conservative attitudes and reluctant intention toward entrepreneurship. The second explanation could apply to graduates of the department of accounting as well. From this perspective, it was understandable that minimal variance in the competencies comparisons was found in the CEP-Y and CEP-N students of the department of accounting. Moreover, regardless of the domain or the CEP-Y or CEP-N classification, the self-assessed scores of each entrepreneurial competency and intention of the students of the department of accounting were the lowest among the departments of the college of management.

It is evident that other factors, such as professional skills trained or the context conditions of employment, could contribute to variations in the effectiveness of the CEP courses on entrepreneurial intention. It is recommended that these findings be referenced for future studies.

### Implications for Practice

#### Suggestion 1: Design to Improve the Effectiveness of CEP for Students Trained With Professional Skills Inconsistent With Entrepreneurship

In terms of education design, this study raises a profound issue: Should CEP equally take all students from any department of the college of management, or should it selectively take those who will demonstrate greater effects from the courses, considering the limited resources of CEP? For example, this study found that students of the department of accounting were the least affected group among the four departments of the college of management. The disciplinary and compliancy values of the professional knowledge and skills of accounting present fundamental conflicts with the hidden core value of entrepreneurship, innovativeness. Taking the inconsistency in the core values of specific professions and the entrepreneurship of CEP into account, should the design of CEP include a special path for students from departments like the department of accounting to enhance the effectiveness of the CEP courses? This is a question for those who plan and execute the strategies of CEP.

#### Suggestion 2: Maximize the Effectiveness of CEP by Selecting Students With Highly Trained Professional Skills

The CEP courses were designed to provide connectivity and the consolidation of knowledge and skills acquired in the regular department courses. The notable effectiveness on students of the department of finance led to the observation that strong professional skills lead to better effectiveness of the CEP courses in the skills domains. A possible explanation is that the students’ professional knowledge and skills provide the fundamentals of effective learning in the CEP classrooms. Therefore, the strong focused professional skills of the department of finance were further enhanced by the CEP courses providing connectivity and consolidation. A contrary case was found for the students of the department of international business, whose professional skills diverged from the course map of the department. Their improvements in the scores of the skills domains provided by the CEP courses were not significant. Therefore, profoundly trained professional skills could be a preliminary condition for the strengthening of skills in the CEP courses.

#### Suggestion 3: The Implementors of the CEP Courses Should Be Aware of the Results of This Study and Modify Their Requirements for Enrolled Students so as to Increase the Effectiveness of Their Entrepreneurship Coaching Efforts

The implementors of the CEP courses should be aware of the results of this study to understand how the effectiveness of their efforts varies in students from the four departments of the college of management. Such understanding should help the implementors to modify their requirements for enrolled students so as to enhance the effectiveness of their coaching efforts for higher entrepreneurship.

The results presented in this study should guide CEP system designers in orienting their efforts on a framework for more effectively consolidating students’ learned professional knowledge and skills, as well as for providing greater encouragement of students’ attitude domains. As a result, the new type of entrepreneurial program, CEP, will better facilitate the entrepreneurship competencies of students with training from traditional management courses.

## Author Contributions

All authors have contributed equally to the conception and design of the study, organized the database, performed the statistical analysis, wrote the first draft of the manuscript. All authors approved the final version of the manuscript.

## Conflict of Interest Statement

The authors declare that the research was conducted in the absence of any commercial or financial relationships that could be construed as a potential conflict of interest.

## Footnotes

1 There are two reasons: (1) Information Management is not viewed as part of the traditional management knowledge domain regarding corporate functions; (2) the Department of Information Management of NTU has been placing more emphasis on information technology than on management, so it will soon be dissociated from the College of Management and merged into the Department of Computer Science and Information Engineering, College of Electrical Engineering and Computer Science, NTU.

## Appendix

**TABLE 1:** The entrepreneurial competencies and intention scale.

**Main theme**	**Sub theme**	**Description**
Knowledge and skills	Knowledge	The professional management courses are useful for starting a business
	Marketing skills	My qualification has provided me with sufficient marketing skills to start a business Marketing skills include conducting market research, assessing the marketplace, marketing products and services, persuasion, getting people excited about your ideas, dealing with customers, communicating a vision, and so on
	Learning skills	My qualification has provided me with sufficient learning skills to start a business Learning skills include active learning, adapting to new situations, coping with uncertainty, and so on
	Interpersonal skills	My qualification has provided me with sufficient interpersonal skills to start a business Interpersonal skills include leadership, motivating others, managing people, listening, resolving conflict, socializing, and so on
	Resource skills	My qualification has provided me with sufficient resource skills to start a business Resource skills include creating a business plan, creating a financial plan, obtaining financing, securing access to resources, and so on
	Opportunity skills	My qualification has provided me with sufficient opportunity skills to start a business Opportunity skills include recognizing and acting on business opportunities and other kinds of opportunities, product/service/concept development skills, and so on
	Strategic skills	My qualification has provided me with sufficient strategic skills to start a business Strategic skills include goal setting, focusing on goals, defining a vision, developing a strategy, identifying strategic partners, and so on
Attitude	Innovativeness	I am a rule breaker
	Self-insight	Amongst various options, I would rather be an entrepreneur
	Entrepreneurial passion	A career as an entrepreneur is totally attractive to me
	Entrepreneurial identity	Being an entrepreneur implies more advantages than disadvantages to me
Intention	Entrepreneurial intention	I will make every effort to start and run my own business
